# Dystocic Labor and Adrenergic and Noradrenergic Neurotransmitters: A Morphological Experimental Study

**DOI:** 10.3390/ijms231911379

**Published:** 2022-09-27

**Authors:** Antonio Malvasi, Antonella Vimercati, Ilaria Ricci, Nico Picardi, Ettore Cicinelli, Ioannis Kosmas, Giorgio Maria Baldini, Andrea Tinelli

**Affiliations:** 1 Department of Biomedical and Human Oncological Science (DIMO), Unit of Obstetrics and Gynecology, University of Bari, 70121 Bari, Italy; 2 Department of Obstetrics and Gynecology, Ioannina State General Hospital G. Hatzikosta, University of Ioannina, 451 10 Ioannina, Greece; 3 Momò Fertilfe Clinic, 76011 Bisceglie, Italy; 4 Department of Obstetrics and Gynecology, and CERICSAL (CEntro di RIcerca Clinico SALentino), Veris delli Ponti Hospital, 73020 Scorrano, Italy; 5 Department of Obstetrics and Gynecology, Second Affiliated Hospital of Xi’an Jiaotong University, Xi’an 710049, China

**Keywords:** dystocia, catecholaminergic neurotransmitters, labor, delivery, adrenaline, epinephrine, noradrenaline, norepinephrine, cesarean section

## Abstract

Authors investigated the catecholaminergic neurotransmitters (chNs) quantitative modifications in pregnant uterine Lower Uterine Segment (LUS) during prolonged labor (PL) with the fetus in an occiput-posterior position (OPP), in occiput transverse position (OTP) and in fetal head asynclitism, all diagnosed by Intrapartum Ultrasonography (IU). The chNs neurotransmitters, particularly adrenaline (or epinephrine-A) and noradrenaline (or norepinephrine-N), were evaluated in LUS fragments sampled during CS of 34 patients undergoing urgent cesarean section (CS) in PL, compared to chNs fibers in the LUS of 36 women submitted to elective CS. All results were statistically analyzed to understand the differences in neurotransmitters morphological analysis by scanning electronic microscopy examination (SEM). The LUS fragments analysis revealed a reduction of A and N fibers in LUS during PL, compared with the expression of A and N fibers in LUS during elective CS. The PL for OPP, the OTP and asynclitism, all positions causing dystocia in labor lead to a reduction in neurotransmitters in LUS, with a uterine vascularization modification and a reduction in the contractility of smooth uterine cells. The A and N neurotransmitters reduction observed in PL negatively interferes with uterine contraction during labor.

## 1. Introduction

Prolonged labor (PL) causes anatomical lower uterine segment (LUS) modification because the fetal head leads to overdistention and vascular modifications in the LUS. This leads to a subsequent phenomenon of vasoconstriction, with tissue ischemia and consequent apoptosis and damage of the neurofibers and neurotransmitters [1]. These anatomical changes during the second stage of labor are particularly evident when the fetal head engages the birth canal in an Occiput Posterior Position (OPP) [2], Occiput Transverse Position (OPT) and asynclitism [3]. In recent years, PL diagnosis with Intrapartum Ultrasonography (IU) has improved the diagnosis of fetal head malposition, therefore the obstetricians can evaluate the best method of delivery for pregnant women to avoid a delay and possible maternal–fetal complications. PL with the fetal head in the birth canal can cause also microscopic modifications of neurotransmitters and neurofibers. Uterine myocytes show a rich innervation by structures of the Autonomous Nervous System (ANS), mainly represented by sympathetic elements. These components play an important role in smooth myocytes’ contractions, more present during labor [4]. The uterus is particularly rich in both adrenergic and cholinergic fibers and these neurotransmitters are involved in the functioning of these microscopic structures as uterine myocytes [5]. Moreover, adrenergic functions of the sympathetic system, mediated by Adrenaline (A) and Noradrenaline (N), are involved in the uterus functionality during labor. The aim of the study was to investigate the difference in neurotransmitters morphological analysis in LUSs of women undergoing PL and non-PL, in terms of quantity of adrenergic and cholinergic neurofibers, in order to understand whether PL for OPP, the OTP and asynclitism, all causes of dystocia in labor, lead to a reduction in the presence of neurotransmitters in LUS and therefore to modifications in the functionality of the LUS.

## 2. Results

Nerve fibers containing adrenaline (or epinephrine-A) and noradrenaline (or norepinephrine-N) were sampled from the LUS of two groups of patients. The two groups were similar in regard to their demographic characteristics (see Table 1).

The results showed a significant reduction in neurotransmitters in the LUS of 36 patients submitted to CS for PL, vs. 34 patients scheduled for elective CS: 10 ± 2.2 vs. 14 ± 1.7, and 9 ± 2.3 vs. 12 ± 1.3, respectively. Statistical analysis demonstrated significantly reduced content of nerve fibers containing adrenaline (or epinephrine-A) and noradrenaline (or norepinephrine-N) in the LUS tissue from elective CS in comparison with those of LUS tissue from urgent CS for PL (*p* < 0.05). All these results are shown in Table 2.

## 3. Discussion

Catecholamines play an important role in uterine contraction and in uterine vascularization setting [6,7]. In fact, A in labor excites contractions of the smooth uterine cells and precapillary sphincteric vessel. Segal et al. [8] investigated the effect of maternal catecholamines on gravid uterine micro vessels caliber. Their results showed that A, detected in the plasma of women in labor, vasodilates uterine resistance vessels and attenuates NA-induced vasoconstriction.

Sean Leonard et al. [9] detected that gestational modification of murine spiral arteries does not reduce their drug-induced vasoconstrictive responses in vivo. Story et al. [10] examined the effects of A, isoprenaline and forskolin upon the evoked contractions of field-stimulated preparations of human, pregnant, isolated myocytes. They enhanced the myometrial contractions of all investigated samples, with a predominant alpha- and not beta-adrenoceptors in a pregnant uterus. Lederman et al. [11] evaluated plasma A and NA concentrations during labor and immediately after delivery, comparing them to those of third trimester uncomplicated pregnancies. Their values returned to normality within 3 to 21 min after delivery and N values remained high or continued to rise in this time interval, also influencing plasma catecholamine levels.

Bengtsson et al. [10] underlined that A and N stimulate myometrial activity in humans, despite clinical doubts about their physiologic and therapeutic importance. During pregnancy, oxytocin progressively stimulates myometrial activity, particularly toward the end of pregnancy. Oxytocin is often used to initiate and stimulate labor. Endogenous A and N can interfere with the pulsatilty of oxytocin, thus affecting the regular uterine contraction and myometrial activity. Ekesbo et al. [12] suggested that the uterine blood flow is regulated by complex interactions of factors, some occurring in nerve terminals and some being circulating humoral factors. Stjernquist et al. [13] demonstrated the existence of NPY in adrenergic nerve fibers surrounding the human uterine artery. A close co-operation between NPY and N in the neuronal control of smooth muscle is suggested. Wikland et al. [14] affirmed that A, N and dopamine all had a stimulatory effect on the uterine contractility; prostaglandins could have a role in the regulation of human catecholamines.

Sato et al. [15] showed that blood flow is regulated reciprocally by parasympathetic vasodilators, mainly via the activation of muscarinic cholinergic receptors, and by sympathetic vasoconstrictors via the activation of alpha-adrenergic receptors. Contraction is produced by the activation of both parasympathetic and sympathetic nerves via muscarinic cholinergic receptors. Fried et al. [16] detected that N and NPY also coexist in human uterine nerves, and that both decrease significantly during pregnancy. Steele et al. [17] evidenced that in pregnancy, human uterine radial arteries are more sensitive to N than during the nonpregnant state. This increase is countered by an endothelium-derived relaxing factor. The factor is unlikely to be nitric oxide.

Weiner et al. [18] underlined that during pregnancy, vascular reactivity of the uterine artery was characterized by decreased stimulation by norepinephrine and increased relaxation by acetylcholine. Toda et al. [19] evidenced that NA from adrenergic nerves contracts venous smooth muscle possibly via the stimulation of alpha 1-adrenoceptors. Segal et al. [20] demonstrated that A, in the plasma of women in labor, vasodilates uterine resistance vessels and attenuates N-induced vasoconstriction. Ekström et al. [21] detected that vasopressin, endothelin, oxytocin and N were significantly more potent on the smallest branches than on the main arterial vessels. Kublickiene et al. [22] evaluated the role of endothelium-derived nitric oxide and endothelin 1 in the modulation of myogenic tone, NE-induced tone, and flow-mediated responses in resistance arteries from pregnant women at term.

Nelson et al. [23] showed that human uterine arteries respond to electrical field stimulations with contraction and relaxation and that these responses may be mediated, respectively, by norepinephrine and, in part, by nitric oxide, released from periarterial nerves. The decrease in neuronally mediated uterine arterial constriction and the increase in dilation could be physiological mechanisms for ensuring appropriate uteroplacental perfusion. Allen et al. [24] highlighted that sympathetic control of cervical blood flow may be modulated by peptide neurotransmitters such as vasoactive intestinal polypeptide and substance P and by local synthesis of prostaglandins E2 and I2. Rakitskaia et al. [25] studied catecholaminergic fiber distribution and N levels studied in sections of the myometrium of a pregnant uterus. They found that catecholaminergic innervation of the myometrium is essentially reduced by the end of normal pregnancy and is detectable only in the lower uterine segment (LUS); in labor, this phenomenon is virtually undetectable. Myometrial N content is reduced by 90% by the end of pregnancy and in labor, the mediator level in the LUS being 2.4 times higher than in the body of the uterus. Table 3 reported the main effects of A and N in abnormal prolonged labor on the smooth cells and micro vessels.

**Figure 1:**
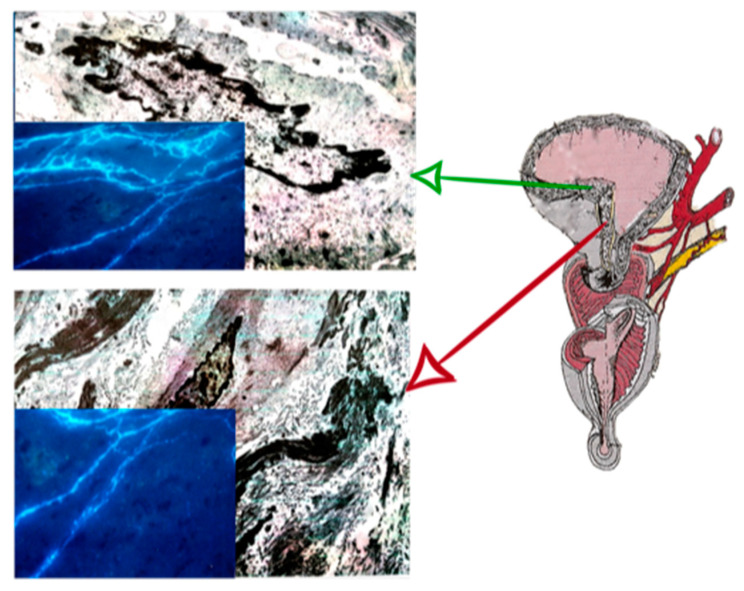
The image shows a sectional drawing of the lower uterine segment (LUS) on the right and the corresponding microscopic photos on the left. These images show, in overlapping, at the electronic scanning microscopy, the LUS with the smooth cell and collagen fibers. The box in blue are the adrenergic (A) and noradrenergic (N) neurofibers: in the box above, the normal LUS in elective cesarean section, in the box below, the urgent cesarean section after prolonged dystocic labor. Note that in the box below, the neurofibers are quantitative less than the neurofibers of the box above.

The reduction in A and N fibers modified the mechanism of uterine smooth cells contractility and micro vessels vascularization, especially in the LUS during labor (Figure 1).

**Figure 2:**
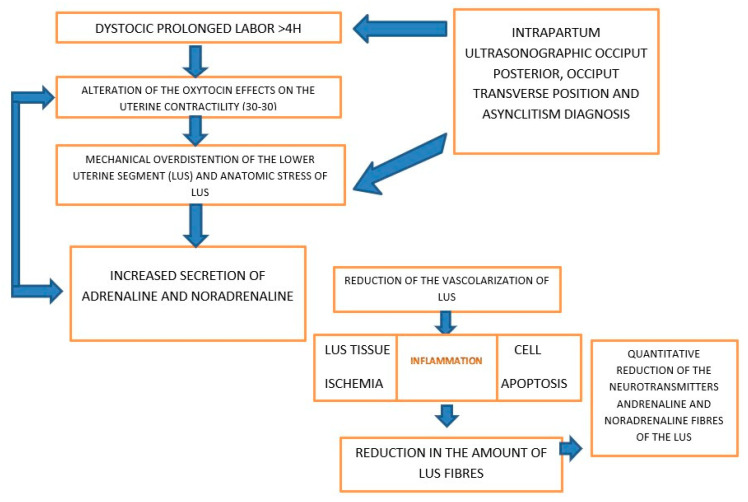
Proposed mechanism of adrenergic and noradrenergic fibers effects in dystocic prolonged labor.

The effects of A and N fibers reduction in PL induce the alteration of uterine contraction, negatively influencing the oxytocin effect on myocytes (Figure 2).

The fetal head in malposition (OPP, OPT and asynclitism) determines an overdistention of LUS, with both muscular, connective tissue and vascular modifications [1]. 

Zuspan et al. [26] evaluated the adrenergic vascularization of the human pregnant uterus, underlying that adrenergic innervation of both the uterus and placenta is preserved during pregnancy.

Tinelli et al. [27] underlined that during labor, major structural and morphological uterine changes occur, especially in the LUS, involving vascular micro-circulation. Obstructed labor can promote tissue ischemia, hypoxia and tissue necrosis, with negative consequences on neuropeptides distribution and function.

Malvasi et al. [28] also affirmed that in dystocic labor, lasting more than 4 h from the complete dilatation, the laminin and collagen IV concentration are reduced in the LUS tissue. In dystocic labor, delivery should be completed before 3 h of full dilation to avoid a reduction in laminin and collagen IV and tissue deterioration of the LUS for the next pregnancy.

In another investigation, authors [29] explained that the presence of neuropeptides reduction in uterine samples of women submitted to urgent CS may be due to a prolonged fetal head station in LUS, with a tissue denervation, as a consequence of both the overdistension and inflammatory process of the dystocic LUS.

Moreover, the same authors [30] reported that collagen IV and laminin show a key role in regulating stiffness of the vesico-uterine space tissue (VUS). The integrity of these substances in VUS tissue is compromised after CS, since wound healing alteration and pelvic pain are related to LUS dysfunctions after CS, as well as pregnancy and delivery complications.

## 4. Materials and Methods

This experimental prospective comparative investigation was performed in southeast of Italy by a collaboration of Department of Biomedical and Human Oncological Science (DIMO), Unit of Obstetrics and Gynecology, University of Bari and the “Veris delli Ponti” Hospital, Dept of Obstetrics and Gynecology, from 2015 to 2021.

The inclusion criteria parameter to be enrolled was pregnant in labor with a singleton fetus in cephalic presentation. The exclusion criteria were the following: women with previous gynecological surgery, fetal macrosomia, infections, VBAC, patients under anticoagulation therapy, toxemia, chorioamnionitis, abnormal invasive placentation, preeclampsia and HELLP syndrome. The control group was composed of patients with a singleton fetus in cephalic presentation to undergo elective caesarean section (CS), for previous term caesarean section, scheduled for diagnosis of breech or transverse presentation, patients with pregnancy after assisted reproductive technique, multiple pregnancies, women wishing to have a caesarean section for personal reasons.

All patients showed a negative vaginal swab (for group B streptococcus, fungi, chlamydia, mycoplasma and other vaginal pathological bacteria). According to the American College Obstetrics and Gynecology guidelines, labor is defined as prolonged labor when it lasts more than 4 h [31]. PL was recorded when the duration of the second stage exceeded 4 h, according to International Guidelines, and showed an angle of progression ≤ 97°. PL for the OPP, the asynclitism or the OPT was diagnosed through vaginal examination (VE) and IU. The OPP was diagnosed by face-to-pubis sign and orbit-to-pubis sign [2]. The asynclitism was diagnosed by the squint sign and asymmetric midline sign [32]. The ultrasound machines involved in IU diagnosis were all GE Voluson E6, equipped with convex probe of 2.7 Mhz. The patients did not receive epidural analgesia during labor and CS was performed in combined spined-epidural anesthesia. Patients also received a prophylactic antibiotic administration of 2 g of Cefazolin intravenously. The surgical technique was a modified Stark’ CS: the uterine incision was transversally on the LUS after bladder flap detachment. After fetus extraction, the placenta was delivered spontaneously, and the uterus was exteriorized. The surgeons sampled four serial consecutive full thickness sections of 5 mm depth (with the inclusion of the myometrial layer) on the LUSs, with the scissors for morphological analysis. Samples included the full thickness of the cervical wall, taken from each LUS using sterile scissors: two samples of approximately 5 mm depth were obtained from the upper and two from the lower edge. The specimens, which were immediately transferred to the laboratory in a dry-ice container, were washed by immersion in a cold Krebs-Ringer’s solution and examined through immunochemical techniques for the detection of cholinergic and adrenergic nerve fibers. The specimens were cut with cryostat to obtain sections of approximately 40 µm. Each section was placed on a cover slide, to which it adhered due to the temperature difference. The cover slides bearing the colored samples were placed on glass microscope slides. Analysis of the samples was carried out with a fluorescence Leitz Ortoplan microscope equipped with an epi-illumination system. The light source was a mercury lamp (HB100) in combination with selective Leitz filters. The density of noradrenergic and of cholinergic fibers was calculated by quantitative analysis using a Quantimet Leitz image analyzer that measures the following parameters: (1) number of adrenaline and of acetylcholine-containing fibers counted in 10 fields randomly chosen; (2) percentage of the total area occupied by those fields; (3) number of observed varicosities; (4) number of crossings or intersections of the nerve fibers; and (5) total perimeter of noradrenaline and of acetylcholine structures in proportion to an average value (100 for each field). Five consecutive serial sections to be checked for LUS were obtained by a transverse section of the upper and lower LUS samples. A 40 nm slides were obtained by cryostat microtome, placed on five separate slides and prepared for the detection of each neurotransmitter. In the first batch: primary or secondary antiserum omitted, denatured or previously absorbed by corresponding peptide in excess; in the second batch: primary or secondary antiserum replaced by a non-immune serum; in the third batch: sample previously fixed by immersion in a 4% solution of formaldehyde in PBS, which did not preserve the immune-reactive sites; in the fourth batch: these samples were denatured with formaldehyde before or after treatment with primary antiserum or before treatment with secondary antiserum.

The samples were incubated for 18 ± 24 h at room temperature due to their thickness, so that the antibodies could completely penetrate the sections. The samples were then washed in PBS and incubated with fluorescing isothiocyanate-conjugated antiserum; the goat anti-rabbit IgG (Nordic Immunological Reagents, Amersham, The Netherlands) diluted 1:100 in PBS for 18 ± 24 h at room temperature allowing the complete penetration of the fluorescent IgG into the slides. Further details on each type of immunoassaying were reported. The fresh cryostatic sections were not mounted during the staining. After the staining had been performed the samples were washed in PBS and then mounted in Entellan (non-auto fluorescent) and examined using a Zeiss III photomicroscope (Carl Zeiss, Oberkochen, Germany), equipped with epi-Illumination and Neofluar objectives. Once the nerve fibers had been stained with specific markers (for acetylcholine and adrenaline), it was possible to identify under light microscopic examination the area of each of the types of nerve fiber stained by a specific fluorescent neurotransmitter. Morphometrical quantification of the density of each type of nerve fiber was performed on photographs of stained samples, using a Quantimet Leica 2000 image analyzer (Quantimet 500 Leica Microsystems Imaging Solutions Ltd., Cambridge, UK). The software provided with the Quantimet Leica analyzer is able to count and express these fluorescent areas in conventional units (C.U.), i.e., as percentages of the area occupied by a single type of nerve fiber in relation to the total observed area. By adding these values (single type of nerve fiber), it is possible to evaluate the sum of the areas occupied by various types of nerve fibers. The software also calculates the average values and reduces them to a single value with standard deviation: this value can be observed on the instrument display and is reported with standard error from the mean (SEM). Other details about the experimental procedures used in the morphometrical quantification by the Quantimet Leica are reported in the Manual of Methods.

Statistical analysis of the data was performed on results obtained from different measurements on each sample; the data was averaged to obtain a median value per case. The mean ± SEM were then calculated and reported for each nerve fiber group. Numerous immune-histochemical controls were made, and the P was calculated as index of significance.

## 5. Conclusions

PL determines anatomical modifications of the gestational uterus, particularly of the LUS. The fetal head in malposition (OPP, OPT and asynclitism) determines a LUS overdistention with vascular modifications, with ischemia and cellular apoptosis. These anatomical changes include alteration in neurotransmission and modifications to uterine contraction activity. 

This study investigated the difference in neurotransmitters morphological analysis in the LUSs of women undergoing PL and non-PL in terms of quantity of adrenergic and cholinergic neurofibers and reported a decrease in quantitative A and N fibers in PL. Therefore, these biological changes occurring during PL determine a significant reduction in the neurotransmitters involved in the mechanism of childbirth, negatively interfering with uterine contraction during labor. All the abnormal fetal head positions causing dystocia in labor can lead to PL, with further problems including the reduction in neurotransmitters in the LUS, with a uterine vascularization modification and a reduction in the contractility of smooth uterine cells, with the uterine contractility reduction and labor pain increasing. 

## Figures and Tables

**Table 1:** Demographic characteristics of the two groups.

	Elective Cesarean Section (*n* = 34)	Cesarean Section in Prolonged Labor (*n* = 36)	*p*
**Age** (year)	34.5 ± 4.1	37.6 ± 3.5	>0.05
**BMI** (kg/m^2^)	28.2 ± 4.7	30.3 ± 1.8	>0.05
**Gestation week** (weeks ± days)	39 ± 5.2	40 ± 4.9	>0.05
**Birthweight** (g)	3230 ± 345	3420 ± 290	>0.05

**Table 2:** Evaluation of A and N nerve fibers in lower uterine segment (LUS) in elective CS and on LUS of patients in prolonged labor submitted to CS.

Group 1: Elective CS (*N* = 34)	Group 2: CS in Prolonged Labor (*N* = 36)	*p*-value
Nerve fibers density containing adrenergic-immune reactivity within specimens of human Lower Uterine Segment (LUS)		
LUS specimens 14 ± 1.7 (immune reactivity)	LUS specimens 10 ± 2.2 (immune reactivity)	<0.05
Group 1: Elective CS (*N* = 34)	Group 2: CS in Prolonged Labor (*N* = 36)	*p*-value
Nerve fibers density containing noradrenaline-immune reactivity within specimens of human Lower Uterine Segment (LUS)		
LUS specimens 12 ± 1.3 (immune reactivity)	LUS specimens 9 ± 2.3 (immune reactivity)	<0.05

**Table 3:** Main effects of adrenaline and noradrenaline on the uterine muscular cells and vessels.

**Adrenaline and Noradrenaline Effects in the Gravid Uterus in Normal Labor (3)**	
Adrenaline (Epinephrine)	Uterine muscle cells: contracting stimulation effect on the uterine smooth cells. Uterine vessels: vasodilatation effect.
Noradrenaline (Norepinephrine)	Uterine muscle cells: contracting stimulation effect on uterine smooth cells. Uterine vessels: vasodilatation effect.
**Adrenaline and Noradrenaline Effects in the Gravid Uterus in Prolonged Dystocic Labor**	
Adrenaline (Epinephrine)	Uterine muscle cells: spastic contraction of the uterine smooth cells (7). Uterine vessels: vasoconstriction (35).
Noradrenaline (Norepinephrine)	Uterine muscle cells: spastic contraction of the uterine smooth cells. Uterine vessels: vasoconstriction.

## Data Availability

The data that support the findings of this study are available from the corresponding author upon reasonable request.

## Abbreviations

ANS	Autonomous Nervous System
LUS	Lower Uterine Segment
IU	Intrapartum Ultrasonography
chNs	Catecholaminergic Neurotransmitters
A	Adrenaline
N	Noradrenaline
SEM	Scanning Electronic Microscopy
PL	Prolonged Labor
DPL	Dystocic Prolonged Labor
OPP	Occiput Posterior Position
VE	Vaginal Examination
OTP	Occiput Transverse Position
